# The Sanitarian

**Published:** 1873-12

**Authors:** 


*The Sanitarian.* A monthly Journal. A. M. Bell, M. D. Editor. Published by A. S. Barnes & Co., N. Y. and Chicago. Annual Subscription $3 00, single number 30 cents.

A perusal of the December number 1873 will show how useful a publication this is, the contents being School House Ventilation, illustrated. The Bermudas, Nature's Scavengers, Artificial Feeding, Proceedings of Am. Public Health Association, etc. etc.

